# FedBranched: Leveraging Federated Learning for Anomaly-Aware Load Forecasting in Energy Networks

**DOI:** 10.3390/s23073570

**Published:** 2023-03-29

**Authors:** Habib Ullah Manzoor, Ahsan Raza Khan, David Flynn, Muhammad Mahtab Alam, Muhammad Akram, Muhammad Ali Imran, Ahmed Zoha

**Affiliations:** 1 James Watt School of Engineering, University of Glasgow, Glasgow G12 8QQ, UK; 2 Department of Electrical Engineering, University of Engineering and Technology, Lahore-Faisalabad Campus, Faisalabad 38000, Pakistan; 3 Thomas Johann Seebeck Department of Electronics, Tallinn University of Technology, 19086 Tallinn, Estonia

**Keywords:** federated learning, artificial neural network, clustering, machine learning

## Abstract

Increased demand for fast edge computation and privacy concerns have shifted researchers’ focus towards a type of distributed learning known as federated learning (FL). Recently, much research has been carried out on FL; however, a major challenge is the need to tackle the high diversity in different clients. Our research shows that using highly diverse data sets in FL can lead to low accuracy of some local models, which can be categorised as anomalous behaviour. In this paper, we present FedBranched, a clustering-based framework that uses probabilistic methods to create branches of clients and assigns their respective global models. Branching is performed using hidden Markov model clustering (HMM), and a round of branching depends on the diversity of the data. Clustering is performed on Euclidean distances of mean absolute percentage errors (MAPE) obtained from each client at the end of pre-defined communication rounds. The proposed framework was implemented on substation-level energy data with nine clients for short-term load forecasting using an artificial neural network (ANN). FedBranched took two clustering rounds and resulted in two different branches having individual global models. The results show a substantial increase in the average MAPE of all clients; the biggest improvement of 11.36% was observed in one client.

## 1. Introduction

With the advent of the Internet of Things (IoT), a significant rise in connected devices and sensors empowered by the 5G communication network generated a large amount of data. The overwhelming availability of data captured by edge devices, coupled with advanced machine learning (ML), inspired many researchers to process data on a massive scale for real-world applications [1]. However, the traditional cloud-centric ML training approach uses centralised data, which requires a large amount of data to be transferred from the end devices to a third-party central server [2]. The data generated by edge devices are privacy sensitive and highly protected under the law [3]. Furthermore, raw data transfer also burdens the communication networks and incurs latency caused by long propagation delays, which is not acceptable in time-sensitive applications [4].

Therefore, to overcome these challenges, a distributed learning approach called federated learning (FL) was introduced. FL aims to train ML models collaboratively, orchestrated by a centralised server [5]. FL is collaborative and continual learning in which a global model is updated by aggregating the local models’ parameters using training on edge devices. In centralised model training, it is assumed that the data are independent and identically distributed (IID). However, due to the decentralised nature of data, this assumption is not applicable in the FL setting. Therefore, federated averaging (FedAVG) is one of the simplest and most commonly used techniques for model aggregation for non-IID data, whereby the weighted average of model parameters is performed on the central server [6]. This model training approach offers privacy by design, because no data are shared.

Short-term load forecasting (STLF) is an essential component of smart grid systems because it allows utilities and grid managers to predict electricity consumption for the coming hours or days. Accurate load forecasting enables better planning and management of resources, which can aid in cost reduction, energy optimization, and grid resilience [7]. Statistical time series techniques such as linear or non-parametric regression, exponential smoothing, auto-regressive moving average, and auto-regressive integrated moving average (ARIMA) are present in the literature [8,9]. However, with the rise of big data and artificial intelligence (AI), more advanced machine learning (ML) algorithms, such as deep neural networks, have been developed to map complicated and non-linear behaviour in historical load profiles. Unfortunately, DL requires the transmission of massive amounts of historical data to a centralised location for model training [10]. Although centralised model training has achieved promising results, it also presents multiple challenges, including data privacy, transmission costs, and secure island data access. Furthermore, the energy market is fiercely competitive, and utility companies are reluctant to share data with their competitors. FL has the potential to solve the challenges mentioned above and train a robust forecasting model without sharing any data.

Despite various advantages, FL has to deal with challenges such as device heterogeneity, privacy, data imbalances, secure aggregation, data diversity, and anomalous clients. For instance, various devices participating in the training process may have diverse computation and storage power, leading to different convergence times. Privacy is often a significant concern in data-driven applications, which FL can solve. However, communicating model updates throughout training can reveal sensitive information to a third party or the central server. This issue is often overcome using homomorphic encryption and differential privacy [11]. Because the server does not have access to the clients’ data, there is the possibility that a client might not be sending proper updates to the server. This problem and its resolution have been discussed in detail in [12]. Similarly, data diversity is one of the biggest challenges in the FL environment. For instance, the model’s accuracy is significantly reduced, by 55%, when it is trained on highly skewed data [13]. Therefore, one possible solution is to share a small amount of data to perform statistical analysis for clients’ clustering on the server. However, data sharing is not feasible due to privacy concerns. Another possible method to address data heterogeneity is clustering the users based on the statistical properties of the model’s parameters on the server. The idea is to obtain multiple global models to improve the system’s performance. In this work, we propose a hidden Markov model (HMM)-based clustering approach, creating multiple global models. The key idea is to monitor each client’s mean absolute percentage error (MAPE) and compute the Euclidean distance of MAPE for each client. The branch on each client represents a separate global model for clients with similar data.

### 1.1. Related Work

STLF involves predicting the electricity demand of a given area over a period of a few hours or days. This is crucial for energy suppliers because it allows them to optimise energy production resources to eliminate power outages and reduce costs. This has traditionally been achieved by collecting data from smart meters to train a centralised model. However, this entails exchanging privacy-sensitive information with a central server, posing serious privacy concerns. Although FL has shown promising results in various applications, there are very limited studies to date on its use for smart grid applications, especially STLF. For instance, the authors of [14] proposed a deep neural network (DNN)-based STLF model using smart meter data. The proposed method uses differential privacy for security, and performance is evaluated by computing the mean absolute percentage error (MAPE). An LSTM-based neural network was proposed for STLF in an agile workspace at the University of Glasgow, UK, with the help of the Persuasive Energy Conscious Network (PECN) [15]. The authors of [9] propose residential energy forecasting using FL and edge computing to ensure the privacy of energy consumption data. The effectiveness of the proposed model is evaluated through experiments using different configurations with varying levels of privacy and compared against a traditional centralised approach. The results revealed that increasing privacy levels typically came at the cost of decreased accuracy in the overall prediction, as demonstrated by the scenarios with different edge-computing configurations. Similarly, a non-clustering-based approach to train long-short-term memory (LSTM) networks for STLF is proposed in [16]. In this study, individual households are used as federated clients, and the results reveal that FL is not suitable for individual households as they have very unusual energy consumption patterns. Additionally, STLF is a complex task that involves many variables, and the effectiveness of FL for this task may depend on the specific characteristics of the data and the energy system being modelled.

In FL, the performance of the global model is highly dependent on the quality of data on each client. Diversity in data significantly impacts the overall performance of the system. The commonly used FedAVG algorithm for model aggregation does not compensate for the divergence in model weights caused by non-IID data. For instance, the authors of [13] analysed the performance of a convolutional neural network (CNN) using non-IID data. The results show a significant divergence between local model weights’ means and standard deviations, resulting in performance degradation. To overcome this challenge, a small subset of global data is created and shared among clients for clustering to improve the model’s accuracy. The authors of [17] proposed an auto-encoder-based clustering approach to group patients’ electronic records into several communities. For each community, a separate model is trained simultaneously based on the similarity of the data, resulting in improved results. A hierarchical clustering approach to deal with non-IID data in FL is proposed in [18]. The idea is to capitalise on the similarity of local updates to create a specialised global model for each subset of clients. This approach not only improves the model’s overall performance, but also reduces the convergence time.

Similarly, the authors of [19] proposed a client clustering approach based on the similarities of gradients computed in local updates. This technique improves the accuracy and communication cost of the system. In [20], a soft clustering algorithm is proposed, using overlapping clusters to cater to the complex nature of the data. The information on overlapping clients can be utilised to categorise similar clients more efficiently to enhance the system’s performance. The authors of [21] proposed FedSim, a clustering approach based on the pairwise similarity of the local gradients. The proposed FedSim algorithm decomposes the training process into local and global steps, aggregating clients with similar gradients to ensure better convergence. The results are compared with those of traditional FedAVG, and the findings of this study confirm that exploiting latent inter-client similarities significantly improves performance.

### 1.2. Motivation and Contributions

With the existing FL mechanism, a single global model is obtained using iterative local updates on the server. However, the data from edge devices are highly diverse, which causes a significant divergence in model parameters, resulting in performance degradation as analysed in [13]. Therefore, multiple global models are the way forward to deal with the diversity of client data. One possible solution is using a global data set for client clustering, which poses serious privacy concerns. Furthermore, federated personalisation is another way of dealing with diverse data, whereby the optimal global model can be aggregated either layer-wise or through model architecture to obtain a personalised model. This personalised training increases the model’s convergence time, as each client needs training for the optimal local model [22]. Another commonly used technique to ensure privacy is based on measuring the similarity of model parameters such as gradient and mean or variance of layerwise weights. The similarity measure is an efficient method of client clustering to deal with data diversity. However, to ensure the privacy of the global aggregation, differential privacy, whereby random noise is added to local weights, is used. With differential privacy, keeping track of similarity measures is challenging, as noise levels will affect model parameters differently [11]. Therefore, to deal with this issue, this work proposes an HMM clustering approach which uses the local loss function MAPE as a metric for measuring the similarity of clients. The aim is to use the Euclidean MAPE distance of each client to develop a branch which represents multiple global models using a subset of local clients. The key contributions of this work are highlighted below:

- We propose a zero-knowledge-based client clustering approach using HMM, which uses the Euclidean distances of loss functions. In each communication round, the clients share the model parameter and value of the loss function, in our case MAPE, to measure the similarity among different clients.
- The proposed FedBranched framework deals with diverse client data in the FL environment to make multiple global models based on the level of data diversity. Furthermore, there is no predefined limit for the number of clusters, which makes this approach more flexible for highly diverse data.
- The use of FedBranched guarantees convergence of the loss function.
- We propose an ANN and selected features for short-term energy forecasting.
- We perform an analysis of energy consumption before and after applying FedBranched.

### 1.3. Paper Organisation

The rest of the paper is organised as follows: Section 2 discusses the proposed framework, and Section 3 explains the simulation setup. Section 4 provides a discussion of the experimental setup and results. The conclusions and future research directions are given in Section 5.

## 2. FedBranched

FedBranched is a zero-knowledge framework that uses HMM clustering to create branches of clients based on the Euclidean distances of MAPE of each client, and each branch is assigned a new global model. The HMM is a generative probabilistic model in which a series of internal hidden states **Z** generates a series of observable **X** variables. Direct observation of the hidden states is not possible. It is assumed that the transitions between hidden states take the shape of a (first-order) Markov chain. The use of HMM in the clustering mechanism is explained in detail in [23].

There can be multiple stages of clustering; however, clustering will only create two branches at any stage. The idea behind restricting the number of branches to two is to reduce the total number of required global models. The maximum number of branches can be n/2, where n is the total number of clients.

At the beginning of FedBranched, a generic ML model is sent to all the clients. When data stored locally are passed through this ML model, the newly generated ML is then called the local model. All the devices send local models and a loss function to the server, and the server aggregates them to make a new model, known as a global model, *M*. We chose MAPE as the loss function, which can be calculated as:

(1) $$\mathrm{MAPE}=\frac{1}{n}\sum_{n}^{t=1}\left|\frac{A_{t}-F_{t}}{A_{t}}\right|$$

where $A_{t}$ is the actual value, $F_{t}$ is the forecasted value, and *n* is the total number of iterations. It is worth mentioning that the MAPE is independent of the system capacity and the unit of measurement. It may be the only error metric that can be used to compare forecasting performance between various utilities [24].

The generation of the global model completes one communication round. After a pre-decided number of communication rounds, the server analyses the MAPE of all clients and identifies non-convergent clients. If the MAPE of all clients does not converge, then the server creates two groups of clients, known as Branch 1 and Branch 2. To make two new branches, the server calculates each client’s Euclidean distances of MAPE and places it in a n∗n matrix. Then, HMM clustering on the sum of each row is used to create two branches. The server now sends a generic ML to both branches, and local branch ML models are sent back to be aggregated into new mini-global models, $M_{1}$ and $M_{2}$. At this stage, there are two global models. This completes one clustering round. Now, the server again checks the convergence of MAPE of all clients in both branches. If the MAPE of any client in any of the branches does not converge, then another round of clustering is required. Branch 1 is divided further into Branches 3 and 4, while Branch 2 is divided into Branches 5 and 6. This only happens if the MAPE of all clients in both branches, 1 and 2, could not converge. If any branches from 1 or 2 converge, then newly gendered branches from non-converging branches will be merged individually with the previously converged branch.

**Figure 1:**
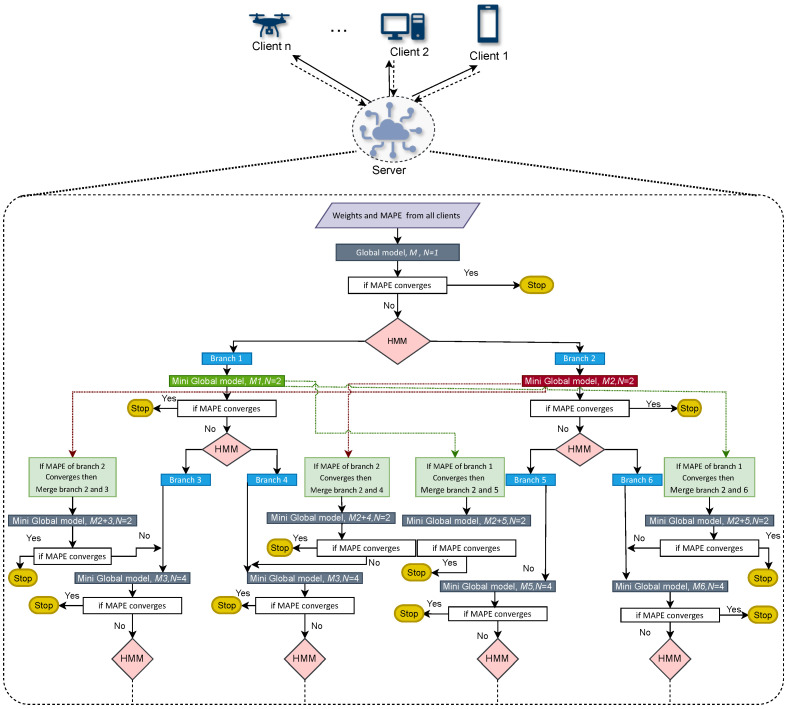
Framework of FedBranched.

Furthermore, the new global model is decided for that branch. For example: the MAPE of all clients in Branch 1 did not converge; however, the MAPE of all clients in Branch 2 has converged. In this case, the client in Branch 1 is sliced into Branches 3 and 4. Now, Branches 3 and 4 are merged with Branch 1 individually, and new mini-global models $M_{1,3}$ and $M_{1,4}$ are created for these branches. If the MAPE of all clients converges in these branches, we will still have two global models. If a MAPE does not converge, we discard $M_{1,3}$ and $M_{1,4}$ and proceed to make global models from Branches 3 and 4, $M_{3}$ and $M_{4}$. Moreover, the branching process continues until all the MAPE of all the clients in all the branches have converged. This process is explained graphically in Figure 1. Here, *N* represents the total number of global models at any stage.

## 3. Simulation Setup

**Figure 2:**
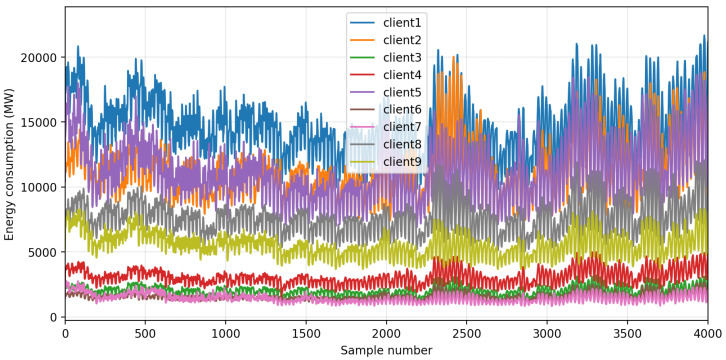
A sample of the data set.

**Figure 3:**
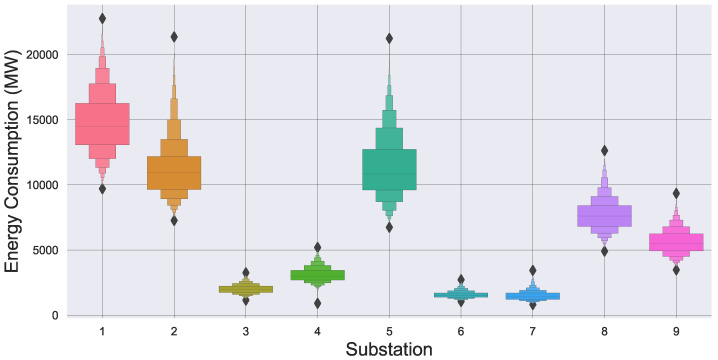
Diversity visualization of the data set.

To test the results of FedBranched and compare them with those of Vanilla FL, we created an FL environment with nine clients. We selected a real-world energy data set from PJM Interconnection, LLC [25]. In this data set, each column represents energy utilised by each substation. A data set sample is plotted in Figure 2. Each client has 13,896 samples. It can be seen that it is a very diverse data set, and the clients do not resemble one another. The letter-value plot in Figure 3 can further confirm the diversity. It can be visualised that Client 1 and Client 7 have the highest and lowest mean, respectively. Furthermore, it can also be noticed that Client 2 has the highest number of outliers. This data set is used for short-term load forecasting (STLF) with the help of five features. The included features were last hour value, last day value, last week value, last 24 h average, and last week average. An artificial neural network (ANN) with three dense layers was designed for STLF. The first layer had one hundred neurons, the second layer had fifty, and the last layer had one neuron. All the layers had relu activation functions and Adam optimiser with mean square error as the loss function. We used a 70/30 split for training and test data. We ran FL for 30 communication rounds; each client had 15 local epochs and a batch size of 300. We selected FedAVG [26] as the aggregation method at the server.

## 4. Experiment and Results

**Figure 4:**
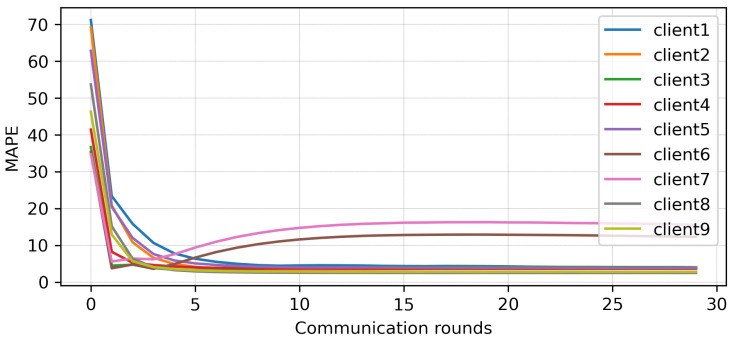
FL results with one global model after 30 communication rounds.

**Figure 5:**
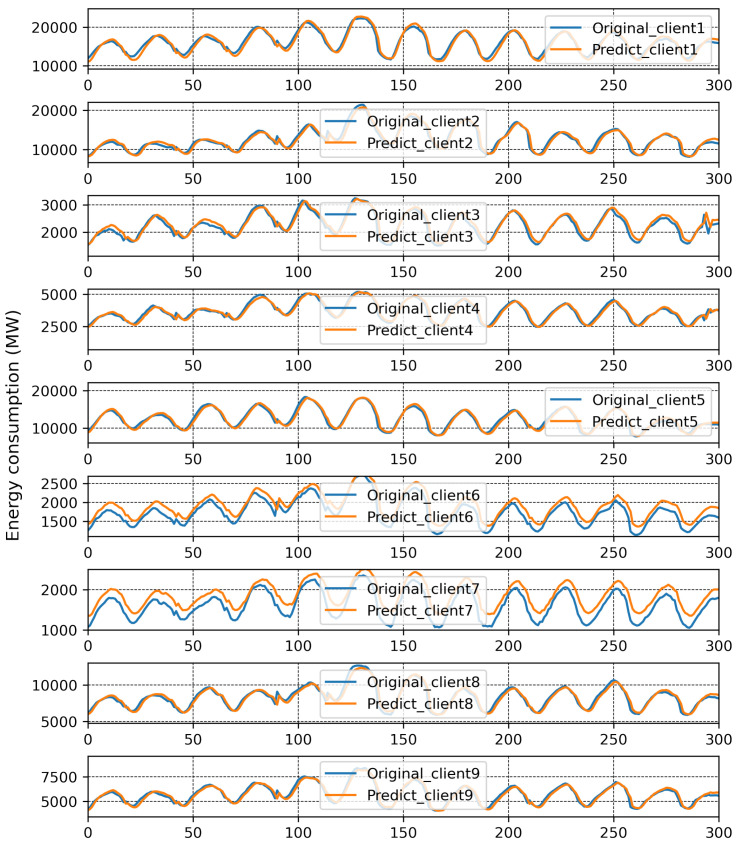
Prediction results with one global model.

The results of traditional FL are plotted in Figure 4, and here, the MAPE of each client from local models with each communication round is reported. It can be seen that the MAPE of all the clients converges at the eighth communication round except for Client 6 and Client 7. The best and worst MAPE, of 2.51% and 15.71%, were produced by Clients 2 and 7, respectively, after 30 communication rounds. It is evident that a single global model is insufficient, and that Clients 6 and 7 should be treated with a separate global model. The original and predicted data of each client is presented in Figure 5. Here, consumed energy is plotted for the sample number. All clients can predict comparatively better than Clients 6 and 7.

### 4.1. Clustering Round 1

**Figure 6:**
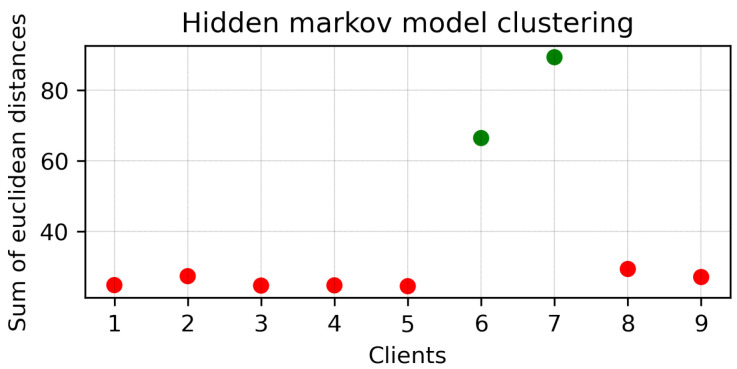
HMM clustering results for round 1. Here, red and green colours are used for different clusters.

**Figure 7:**
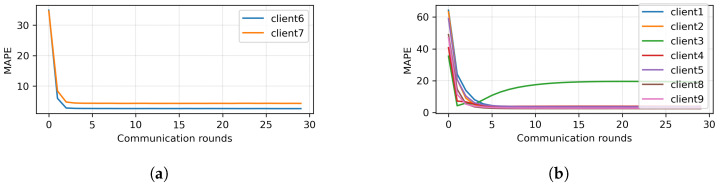
First round of clustering. (**a**) FL Result of Branch 2. (**b**) FL Result of Branch 1.

**Figure 8:**
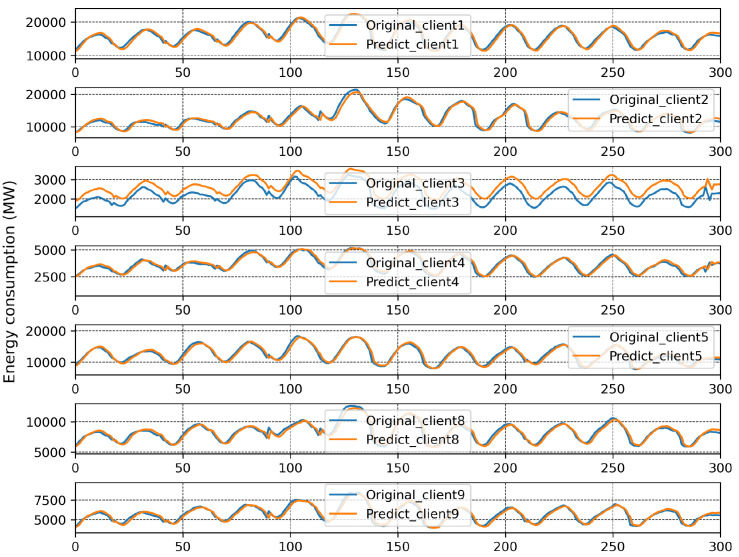
Prediction results of Branch 1.

**Figure 9:**
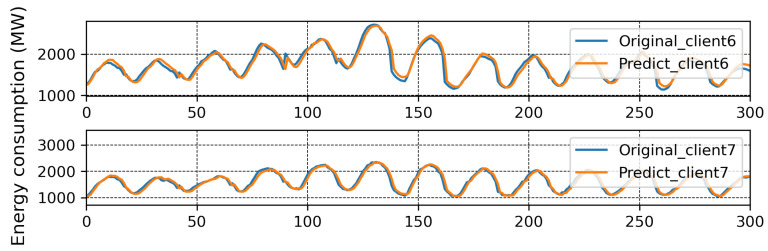
Prediction results of Branch 2.

Because the MAPE of all clients did not converge, HMM clustering was applied to divide the clients into two branches. The results of clustering are presented in Figure 6. Here, Clients 1, 2, 3, 4, 5, 8, and 9 were clustered together (Branch 1), while Clients 6 and 7 were clustered together (Branch 2). Next, two different mini-global models were created for each branch. The MAPE of each client of Branches 1 and 2 are plotted in Figure 7a,b, respectively. It can be seen that, with the MAPE obtained during model aggregation in 30 communication rounds in both branches, the MAPE of all clients except Client 3 from Branch 1 converged. The original and predicted curves are plotted in Figure 8 and Figure 9. In Branch 1, the lowest and highest MAPE, of 2.5% and 18.96%, were obtained from Clients 8 and 3, respectively. In Branch 2, MAPE of 2.6% and 4.3% was obtained from Clients 6 and 7, respectively. At this stage, two mini-global models, $M_{1}$ and $M_{2}$, were created for Branches 1 and 2, respectively. However, because the MAPE of Branch 1 did not converge during model aggregation, we can discard $M_{1}$ and apply HMM for further clustering. It is worth noticing that the MAPE of Client 3 converged when all the clients were grouped during model aggregation, as seen in Figure 4, which means Client 3 performs better with clients in the other branch. Furthermore, it can be observed that Branch 1 required two global models.

### 4.2. Clustering Round 2

**Figure 10:**
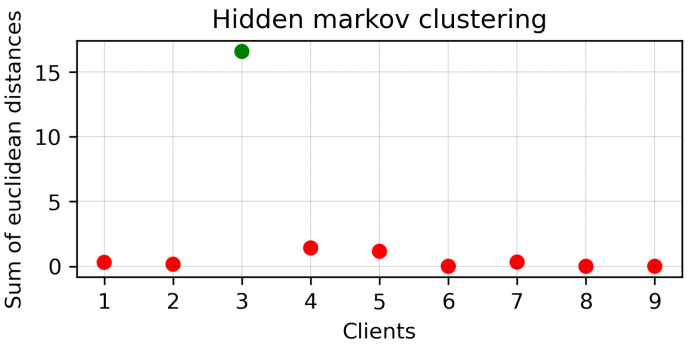
HMM clustering in round 2. Here, red and green colours are used for different clusters.

**Figure 11:**
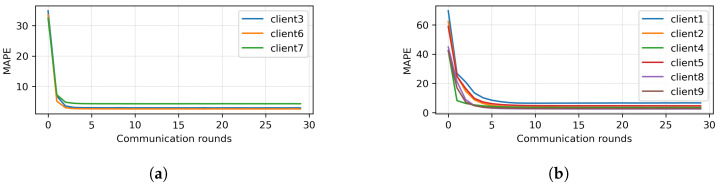
FL results of second round of clustering after 30 communication rounds. (**a**) FL results when Branch 2 + 4. (**b**) FL result of Branch 3.

We applied HMM clustering to Branch 1, and the results are plotted in Figure 10. Clients 1, 2, 4, 5, 8, 9 are placed in Branch 3, and Client 3 in Branch 4. According to the FedBranched framework, before making new global models for Branches 3 and 4, both must be merged with the previously converging branch (Branch 2). When Branch 4 was converged with Branch 2 (Clients 3, 6, and 7) a new global model, $M_{2,4}$, was generated. The MAPE of all clients in Branches 3 and 4 is plotted with respective communication rounds in Figure 11a,b. It can be seen that the MAPE of all clients converged. Because merging Branch 2 and 4 produced converging MAPE during the model aggregation, there is no need to merge Branch 3 with Branch 2 because we cannot place the same clients in two different branches. A new mini-global model, $M_{3}$, was created for Branch 3, having Clients 1, 2, 4, 5, 8, and 9. The MAPE of all clients during model aggregation are plotted in Figure 11b. It can be seen that the MAPE of all the clients converged during model aggregation.

### 4.3. Discussion

**Figure 12:**
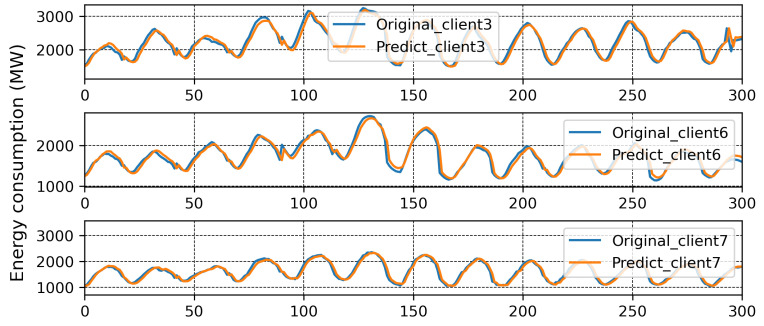
Prediction result when Branches 2 and 4 were merged together.

**Figure 13:**
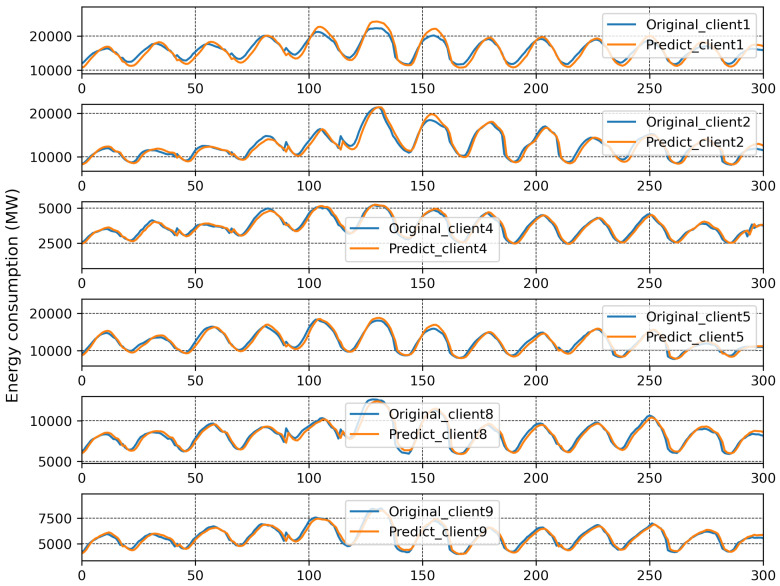
Prediction result of Branch 3.

**Figure 14:**
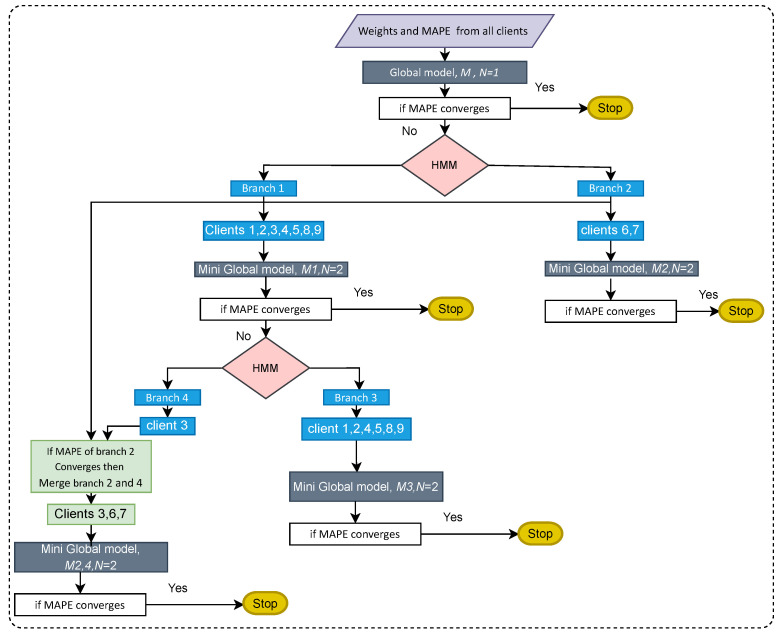
Graphical elaboration of FedBranched on considered example.

After running FedBranched for two clustering rounds, only two mini-global models, $M_{2,4}$ and $M_{3}$, were finalised. The original and predicted curves from both branches are plotted in Figure 12 and Figure 13. The results of traditional FL and FedBranched are summarised in Table 1. The highest percentage improvement, of 11.36%, is observed for Client 7, and the lowest is −0.32%, for Client 4. The clustering mechanism using FedBranched on the given data is graphically elaborated in Figure 14. It can be seen that Clients 3, 4, and 7 were clustered together while Clients 1, 2, 3, 4, 5, 8 and 9 were put into another cluster. For both clusters, individual global models were assigned. Our results suggest that FedBranched can successfully improve the forecasting accuracy of highly diverse data. The average MAPE of all clients using Vanilla FL was 5.172%; after applying FedBranched, it dropped to 2.83%.

## 5. Design Insights and Analysis

In this paper, the authors present a framework that requires zero knowledge of the data set and uses a probability-based clustering approach for heterogeneous data in FL. This framework ensures the convergence of the loss function.

1. **Key features:** FedBranched is capable of clustering clients without looking at the user’s data. Clustering is performed on the sum of Euclidean distances of a loss function using HMM. Here, a multi-stage clustering mechanism is adopted to minimize the required clusters and global models. At any stage, there can be only two clusters. The purpose of restricting the number of clusters is to have control and restrict the number of clusters to a minimum.
2. **Scope:** FedBranched uses loss functions rather than weights of ML models; it can work perfectly with differential privacy [27] and homomorphic encryption [28].
3. **Limitations:** Because it is a clustering-based approach, it might not work well when the number of clients is less than five.
4. **Drawbacks:** FedBranched uses a multistage clustering approach depending upon the diversity of data. At each stage, new branches are created and their respective global models are decided after running FL for pre-decided communication rounds. This will increase the computation power and time required to achieve an optimal number of global models that can satisfy the user’s requirements. In this paper, Vanilla FL was carried out for 30 communication rounds, whereas FedBranched was run for 150 (30*5) communication rounds, as shown in Figure 14.
5. **Energy consumption:** Energy consumption, $E_{com}$, in the training process of a global model depends on many factors, such as energy consumed per kb when local and global data are shared between clients and server, time of data transfer, type of IoT device used, the communication channel used, computation time on each device, etc. Here, $E_{com}$ is described based on the work of [29]:
  (2) $$E_{com}=R\left[\left(\alpha *t\right)+\left(\beta *D\right)\right]$$
  where *R* represents number of communication rounds, α represents energy per second, *t* is for time, β represents energy consumed per kb, and *D* represents amount of data transfer. In our experiments, each global or local model is of 38 kb and β is taken as 0.015 kWh/GB from [30], while α is assumed as 0.0001 kWh/sec; then, baseline $E_{com}$, when *t* is 3.39 min, is equal to 27 W. However, using FedBranched, $E_{com}$ reached 136 W.

## 6. Conclusions and Future Work

The use of diverse data sets in FL can lead to high forecasting error in some local models, which can be categorised as an anomaly. In this paper, the authors have presented FedBranched, a zero-knowledge clustering-based framework to tackle diverse data sets in FL. Clustering is performed using HMM clustering on the Euclidean distance of MAPE obtained after a pre-decided number of communication rounds. This framework divides clients into different branches and assigns their respective global models. Clustering is performed in multiple rounds depending upon the diversity of data, and there can be a maximum of n/2 clusters where n is the total number of clients; however, at any stage, there can only be two branches. The developed framework was applied to a highly diverse, real-life energy data set obtained at the substation level, having nine clients, for STLF using ANN. FL was carried out for thirty communication rounds, and the ML model was trained for fifteen epochs in each round. The initial results of FL showed that one global model is not enough for nine clients. FedBranched took two clustering rounds and ended up dividing nine clients into two clusters and two global models. The use of FedBranched resulted in a noticeable increase in MAPE. The highest improvement of 11.6% was observed for Client 6. However, energy consumption analysis shows that during the training process, the global model Vanilla FL consumed only 27 W, while FedBranched used 136 W. The authors are currently working on an extension of this work, which aims to design an efficient framework that uses all of the possible combinations of clients and transfer FL to make a global model that provides the lowest loss function. This will remove the need for multiple global models. 

## Figures and Tables

**Table 1:** MAPE comparison of traditional FL and FedBranched.

Client	Vanilla FL (%)	FedBranched (%)	Percentage Improvement (%)
1	4.13	2.78	1.35
2	2.80	2.66	0.14
3	4.05	2.98	1.07
4	3.63	3.95	−0.32
5	3.88	3.65	0.23
6	12.43	2.61	9.82
7	15.71	4.35	11.36
8	2.51	2.50	−0.01
9	2.85	2.82	0.03

## Data Availability

The data set used is available at: www.kaggle.com/datasets/robikscube/hourly-energy-consumption (accessed on 8 August 2022).

## Abbreviations

The following abbreviations are used in this manuscript:

FL	Federated learning
ML	Machine learning
n	Total number of clients
N	Total number of global models
M	Global model
ANN	Artificial neural network
STLF	Short-term load forecasting
